# Advantages of the Prone Position in Examining the Fœtal Circulation as a Diagnostic Sign of Pregnancy

**Published:** 1858-02

**Authors:** W. Byford

**Affiliations:** Professor of Obstetrics in Rush Medical College


ARTICLE IV.

### ADVANTAGES OF THE PRONE POSITION IN EXAMINING THE FŒTAL CIRCULATION AS A DIAGNOSTIC SIGN OF PREGNANCY.

BY W. BYFORD, M. D.,

Professor of Obstetrics in Rush Medical College.

The fœtal heart may be heard to beat several weeks sooner by placing the woman on her face, with the abdomen pendant, than in the supine or erect position. In the prone position, with the head lower down than the level of the feet, and the abdomen hanging freely down from the spinal column, the uterus rises out of the pelvis, and, by its weight, keeps in close proximity to the anterior wall of the abdomen; and the fœtus, being of greater specific gravity than the liquor amni, will settle down on the most dependent part of the uterus. In this condition of things, the ear of the observer or the end of the stethoscope is placed against the lowest part of the uterine tumor, and pressed pretty firmly upward against it, and thus brought pretty close to the heart of the contained fœtus, nearer, in fact, than in any other position that can be assumed. In cases of doubt between peritoneal dropsy and pregnancy, or in which an abundance of liquor amni simulates dropsy of the peritoneal sac, this position will avail us in making the diagnosis. As in early pregnancy, when the liquor amni is relatively abundant, the uterus settles down on the abdominal walls by virtue of its weight, while the child will sink to the bottom of the liquor in which it is contained. By this procedure we may diagnosticate with certainty what has hitherto been the most obscure cases, such as have time and again betrayed the most learned and skillful obstetricians into grievous errors of diagnosis and treatment. More than one case are recorded by their candid authors in which tapping diagnosticated the existence of pregnancy independently, or connected with dropsy of the peritoneum. If the child is sufficiently healthy and vigorous to perform its circulating functions properly, there need be no difficulty in detecting the sound of the heart in those embarrassing cases by this method. Although not necessary in ordinary cases, this plan will enable us more easily and readily to discover the sound of the fœtal heart and general motions of the child than if the woman was placed on her back in bed. By it we may also isolate the sounds of the uterine and placental vessels from those of the aorta and iliac arteries. The uterus separated from the spine by settling upon the dependent abdominal muscles, will not conduct the sound made by, because no longer in contact with, these vessels; while the vessels of the uterus and placenta may be heard with great distinctness. Not long since I was enabled to diagnosticate the existence of pregnancy in combination with peritoneal dropsy, and perhaps increased liquor amni, with the patient in the prone position that I could not in the dorsal. Ordinarily, it will be sufficient to cause the patient to stand by, and lean her chest upon a moderately low bed, in order to give us a fair opportunity to examine the abdomen, particularly if pregnancy is somewhat advanced. The flexible stethoscope is a very handy instrument to relieve us from a fatiguing and not very delicate posture. It is indispensable that the patient should have no clothing on the anterior part of the abdomen but the chemise; and if this region is entirely nude, so much the better. In cases requiring a more thorough examination, as in the early periods of pregnancy or complications, the thighs will be in our way, and prevent the requisite freedom of examination. The patient should be placed, in such instances, on an inclined plane, so arranged as to leave the abdomen pendant. This may be made by chairs, stools or tables, with pillows placed upon them. Although I am not aware of it, the above suggestions may have been made by some one else long ago. However this may be, I am satisfied that they will be of great use to many members of the profession, if they will remember them when appropriate cases present themselves in their practice.

